# The efficacy of the novel zinc-containing desensitizer CAREDYNE Shield on dentin hypersensitivity: a study protocol for a pilot randomized controlled trial

**DOI:** 10.1186/s13063-020-04426-8

**Published:** 2020-06-03

**Authors:** Takashi Matsuura, Megumi Mae, Masayuki Ohira, Yasunori Yamashita, Ayako Nakazono, Kouji Sugimoto, Kajiro Yanagiguchi, Shizuka Yamada

**Affiliations:** Department of Periodontology and Endodontology, Nagasaki University Graduate School of Biomedical Sciences, 852-8588, 1-7-1, Sakamoto, Nagasaki, Nagasaki Japan

**Keywords:** Dentin hypersensitivity, Desensitizer, CAREDYNE Shield, Nanoseal, Randomized clinical trial

## Abstract

**Background:**

Dentin hypersensitivity (DH) is a condition characterized by short and sharp episodes of pain which will arise in response to tactile, chemical, thermal, evaporative or osmotic stimuli. The painful symptoms cause discomfort in patients and reduce their quality of life. Recently, the novel zinc-containing desensitizer CAREDYNE Shield has been developed as a new type of desensitizer that acts by inducing chemical occlusion of dentinal tubules, and releasing zinc ion for root caries prevention. However, the clinical effectiveness of CAREDYNE Shield on DH remains unclear. Therefore, the aim of this study is to evaluate the effectiveness of CAREDYNE Shield on DH by comparing with that of another desensitizer, Nanoseal, commonly used in Japan.

**Methods/design:**

This study protocol is a two-arm, parallel, pilot randomized controlled trial. Forty DH patients will be randomly allocated to two groups. Participants in the intervention group will be treated with CAREDYNE Shield, while those in the control group will be treated with Nanoseal. The primary outcome is the reduction of pain intensity in response to air stimuli measured with a 5-point verbal response scale from baseline to 4 weeks after the intervention, and Fisher’s exact test will be used for analyses.

**Discussion:**

CAREDYNE Shield can be casually applied to subgingival areas and proximal surfaces because it reacts with only tooth substance. Furthermore, zinc has been reported to reduce the demineralization of enamel and dentin and inhibit biofilm formation, plaque growth and dentin-collagen degradation. Therefore, CAREDYNE Shield may be expected to be a useful novel desensitizer that acts not only as a desensitizer but also as a root caries inhibitor.

**Trial registration:**

UMIN Clinical Trials Registry (UMIN-CTR), ID: UMIN000038072. Registered on 21 September 2019.

**Trial status:**

This study (protocol version number: version 1.4.0; approved on 22 October 2019) is ongoing. The recruitment of participants began in December 2019 and will be continued until November 2020 (Hanke, Am Dent Assoc 27:1379–1393, 1940).

## Background

Dentin hypersensitivity (DH) is a condition characterized by short and sharp episodes of pain which arise in response to tactile, chemical, thermal, evaporative or osmotic stimuli, and which cannot be ascribed to any other form of dental defect or pathology [1, 2]. A recent systematic review reported the DH prevalence in various population to range from 1.3 to 92.1%, and the estimated DH prevalence analyzed in a random-effects meta-analysis was 33.5% (95% confidence interval 30.2–36.7%) [3]. The painful symptoms cause discomfort in patients and reduce their quality of life [4].

Normally, dentin is covered by enamel or cementum and is not affected by direct stimuli; however, once the dentin has been exposed and dentinal tubules are patent in the oral environment, the painful symptoms of DH arise in response to external stimuli. Dentin exposure results from a loss of enamel or cementum. The loss of enamel can be attributed to erosion, abrasion or abfraction, while the loss of cementum is caused by gingival recession associated with improper tooth-brushing, periodontal disease and periodontal surgery.

A number of theories have been suggested to explain the mechanisms of DH; however, the hydrodynamic theory has been widely accepted [5, 6]. According to this theory, fluid movement in the patent dentinal tubules occurs in response to external stimuli, stimulating sensory nerve endings located at the dentin-pulp interface. Thus, dentinal tubule occlusion, which reduces the fluid movement in the dentinal tubules, is one of the ideal DH treatments and is performed with adhesive systems or desensitizing agents that form insoluble mineral precipitates in the patent dentinal tubules [7].

One of desensitizing agents which form insoluble mineral precipitates in patent dentinal tubules is the fluoroaluminocalciumsilicate-based desensitizer Nanoseal (Nippon Shika Yakuhin, Yamaguchi, Japan). The components are similar to those of silicate cement. Nanoseal acts as a desensitizer by a chemical reaction resulting in insoluble nanoparticles that aggregate on the tooth surface for dentinal tubule occlusion and has been suggested to protect the root surface from demineralization via ions, such as calcium or fluorine, released from Nanoseal [8–10]. Thus, Nanoseal may act not only as a desensitizer but also as a root caries inhibitor.

Recently, the fluorozinccalsiumsilicate-based desensitizer CAREDYNE Shield (GC Dental Industrial Corporation, Tokyo, Japan) has been developed. It acts as a desensitizer by inducing chemical occlusion of dentinal tubules and contains a novel functional filler that releases not only ions, such as calcium and fluorine, but also zinc. Zinc has been reported to reduce the demineralization of enamel and dentin, and inhibit dentin collagen degradation, plaque growth and biofilm formation [11, 12].

However, the effectiveness of CAREDYNE Shield on DH still remains unclear; therefore, the aim of this study is to investigate the effectiveness of CAREDYNE Shield on DH by comparing with that of Nanoseal. The PICO question of this study is described in Table 1.

**Table 1:** PICO question

**Criteria**	**Description**
P (participants)	Non-carious human permanent teeth with DH
I (Intervention)	DH treatment with CAREDYNE Shield
C (Control)	DH treatment with Nanoseal
O (Outcome)	The reduction of pain level in response to air stimuli

*DH* dentin hypersensitivity

## Methods/design

### Trial design

This study protocol is a two-arm, parallel, pilot randomized controlled trial; that was developed in accordance with the Standard Protocol Items: Recommendations for Interventional Trials (SPIRIT) and Consolidated Standards of Reporting Trials (CONSORT) guidelines [13, 14]. A SPIRIT Checklist is attached in Additional file 1.

### Participant timeline

**Fig. 1:**
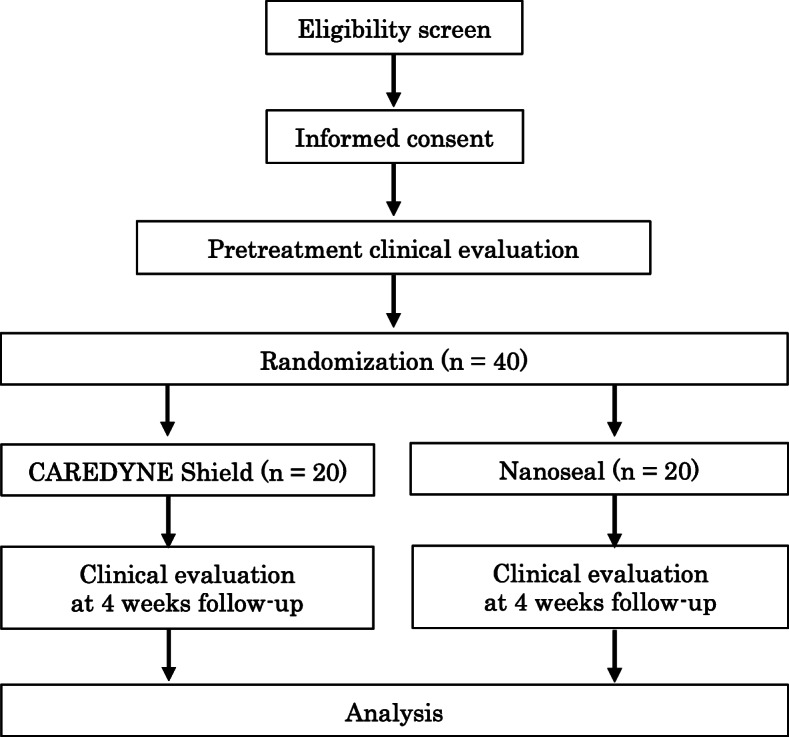
Consolidated Standards of Reporting Trials (CONSORT) flow chart

To describe the time schedule of enrollment, intervention and assessment, a SPIRIT Figure and a CONSORT flow chart are presented in Table 2 and Fig. 1.

**Table 2:** Standard Protocol Items: Recommendations for Interventional Trials (SPIRIT) Figure

	**Enrollment**	**Post allocation**
**Time point**	0	4 weeks (range 3–5 weeks)
**Enrollment:**		
Eligibility screen	X	
Informed consent	X	
Allocation	X	
**Intervention:**		
CAREDYNE Shield	X	
Nanoseal	X	
**Clinical assessment:**		
Air blow	X	X
Inspection	X	X
Palpation	X	X

### Study setting

All procedures of this study will be performed at the Department of Periodontology and Endodontology, Nagasaki University Graduate School of Biomedical Sciences, Nagasaki, Japan.

### Sample size

No previous clinical studies have investigated the effectiveness of CAREDYNE Shield on DH; therefore, at least 15 participants will be required in each group to perform a sample size calculation in a subsequent study [15]. With a 20% dropout rate, a total of 40 participants will be recruited in this study.

### Eligibility screen

When patients present with DH complaints at Nagasaki University Hospital, clinical diagnosis will be performed by the dentist in charge of the patient. Short and sharp pain episodes of pain which arise in response to air stimuli or tactile stimuli will be assessed with a three-way dental syringe or a dental explorer, caries or restoration will be assessed with a dental mirror and a dental explorer, severe periodontal diseases will be assessed with a dental mirror and a periodontal probe and the information about medical/dental history will be obtained by interview. Then, the eligibility criteria described in Table 3 will be verified.

**Table 3:** eligibility criteria

**Inclusion criteria**	
Outpatients	
Participants who presented with a DH complaint	
Participants who agreed to participate in this study after providing their informed consent	
**Exclusion criteria**	
Participants who have an allergy to the desensitizing materials used in this study	
Participants who are pregnant or lactating	
Participants who have undergone DH treatment within the last 6 months	
Participants with systemic diseases that might influence the results of this study	
Participants who present with pain complaints that might influence the results of this study	
DH teeth with restoration that might influence the results of this study	
DH teeth with caries or advanced periodontal disease	
DH teeth that have undergone periodontal surgery or orthodontic treatment within the last 3 months	

*DH* dentin hypersensitivity

### Informed consent

After eligibility screen, the potential participants who met the eligibility criteria will be asked for their informed consent using the informed consent form. They will be then enrolled in this study by an examiner blinded to the allocation. A signed informed consent form is mandatory for enrollment.

### Randomized allocation

After enrollment, baseline assessments will be performed by the examiner who enrolled the participant in this study. Randomized allocation will then be performed in a 1:1 ratio by the dentist in charge of the patient with opaque, sealed envelopes prepared before participant recruitment and on which “Nanoseal” or “CAREDYNE Shield” is printed. Participants allocated to the intervention group will be treated with CAREDYNE Shield, while those allocated to the control group will be treated with Nanoseal. The information on the desensitizers used in this study is described in Table 4.

**Table 4:** Desensitizers used in this study

**Material**	**Manufacturer**	**Composition**
CAREDYNE Shield	GC Dental Industrial Corporation, Tokyo, Japan	Solution A: fluorozinccalciumsilicate glass Solution B: 10–15% phosphoric acid
Nanoseal	Nippon Shika Yakuhin Co., Ltd., Shimonoseki, Japan	Solution A: fluoroaluminocalciumsilicate glass Solution B: 10% phosphoric acid

### Allocation concealment

After the allocation, the operator who performed allocation will record the operator’s name, baseline date and type of teeth to be treated in the allocation list. The list will not contain the allocated group name in order to conceal the allocation as well as the participant’s name and ID number for personal information protection. Allocation information will be recorded in the electronic medical record system which research staff cannot check without leaving a record of their browsing history.

### Blinding of participants

Participants will be blinded during this study and disclosure of allocation to participants will be performed in the following cases: (1) participant requests to stop or change the allocated intervention, (2) worsening disease or new disease occurs, (3) continuing this study is judged to be inappropriate for the participant or (4) this study is terminated.

### Intervention

After randomized allocation, the participants in the intervention group will be treated with CAREDYNE Shield, and those in the control group will be treated with Nanoseal by the operator who performed allocation. Prior to the application of CAREDYNE Shield or Nanoseal, dental prophylaxis and water rinse will be performed to remove plaque deposits; the areas to be treated will be isolated with cotton rolls and dried with cotton pellets; two equal proportions of solution A and solution B will be mixed with a micro-brush and applied to the dentin surface for 20 s, followed by rinsing with water. Four weeks after the treatment, clinical assessments will be performed by the examiner who enrolled the participant in this study.

During this study, any other dental treatment to DH teeth are prohibited, and all intervention procedures will be recorded by the operator in an electronic chart to improve adherence to intervention protocols. Discontinuing intervention will be performed in the following case: (1) participant requests to stop or change the allocated intervention, (2) worsening disease or new disease occurs or (3) continuing this study is judged to be inappropriate.

### Primary outcome

The primary outcome is the reduction of pain intensity in response to air stimuli measured with a 5-point verbal rating scale (VRS) from baseline to 4 weeks after the intervention. To evaluate the pain level, an air blast will be applied using a three-way dental syringe after the isolation of the DH teeth with cotton rolls, participants will then be asked verbally to rate the level of pain intensity using a 5-point VRS (numerical scale from 0 to 4 summarized in Table 5).

**Table 5:** Verbal rating scale

**Score**	**Level of pain intensity**
0	No pain
1	Mild pain
2	Moderate pain
3	Severe pain
4	Extremely intense pain

### Secondary outcome

Secondary outcomes are the change in the gingival condition near the treated area measured with the Gingival Index (GI) and the change in the oral hygiene status at the treated dentin surface measured with the Plaque Index (PI) from baseline to 4 weeks after the intervention [16, 17]. The GI and PI will be evaluated with inspection and palpation.

### Data collection

Outcomes will be assessed at baseline and 4 weeks after the intervention by the examiner who enrolled the participant in this study. Calibration was performed to promote the data quality. The examiner’s name, date of assessment, type of teeth and the acquired outcomes will be recorded in the assessment form and it will be given to the lead principal investigator.

### Data management

Double data entry will be performed by two research staff independently. To ensure confidentiality, all documents obtained in this study will be kept strictly in a lockable filing cabinet in the office for at least 5 years after this study, before being destroyed using a shredder and discarded.

### Statistical design

The primary outcome will be analyzed with Fisher’s exact test according to the intention-to-treat principle. Participants who discontinue or deviate from the intervention protocols or with any missing data will be excluded. No additional analyses will be performed.

### Access to data

The principal investigator will have access to the final study data and make the final decision to terminate this study. No other research staff will have access to any data acquired in this study.

### Monitoring

Monitoring will be performed by one of our research staff according to the standard operating procedures and the results will be given to the principal investigator within 2 weeks after monitoring. When adverse events or other unintended effects of the intervention happen, the principal investigator will perform appropriate treatments for the participant, report the incident to the Nagasaki University Hospital Clinical Research Ethics Committee (REC) and share this information with the research staff. A data monitoring committee is not necessary because DH treatment with Nanoseal or CAREDYNE Shield is general practice and a low-invasive intervention procedure.

### Potential benefits and harms

This study will contribute to future clinical improvements. However, atopic dermatitis is a potential side effect of the intervention.

## Discussion

Several limitations associated with the present study warrant mention. First, blinding of operators will not be performed; therefore, the risk of bias in this domain may be unavoidable. Second, the data will be collected just after the participant’s training. Thus, VRS which is easier to understand for participants than a visual analog scale (VAS) will be performed; however, VRS is considered to be less sensitive than VAS [18, 19]. Third, it has been recommended that at least three different stimuli, specifically tactile, cold and air stimuli, should be used for clinical assessments of DH because DH may differ among stimuli [2]. However, only air stimuli will be evaluated in the present study because this study is being performed for sample size calculations, which is based on the primary outcome: the reduction of pain intensity in response to air stimuli. Tactile stimuli will be evaluated as a secondary outcome in a future, large-scale study. The GI and PI will be used as secondary outcomes in the present study. If a reduction in either is observed, the effectiveness of CAREDYNE Shield for the reduction of the GI and PI will be investigated in a future clinical study.

Desensitizers like CAREDYNE Shield and Nanoseal that induce chemical occlusion of dentinal tubules are biocompatible and react only with tooth substance; they can, therefore, be casually applied to subgingival areas and proximal surfaces. Furthermore, for several decades, in-vitro studies have investigated the function of zinc and reported that zinc reduced the demineralization of enamel and dentin and inhibited dentin collagen degradation, plaque growth and biofilm formation [20–24]. Thus, CAREDYNE Shield, which releases zinc ions, may be a useful novel desensitizer that acts not only as a desensitizer but also as a root caries inhibitor.

## Supplementary information

**Additional file 1.** Standard Protocol Items: Recommendations for Interventional Trials (SPIRIT) 2013 Checklist: recommended items to address in a clinical trial protocol and related documents.

## Data Availability

Not applicable

## Abbreviations

CONSORT: Consolidated Standards of Reporting Trials
DH: Dentin hypersensitivity
RCT: Randomized clinical trial
REC: Research Ethics Committee
SPIRIT: Standard Protocol Items: Recommendations for Interventional Trials
UMIN-CTR: The University Hospital Medical Information Network-Clinical Trials Registry
